# Thermal influence on development and morphological traits of *Aedes aegypti* in central India and its relevance to climate change

**DOI:** 10.1186/s13071-025-06924-7

**Published:** 2025-07-11

**Authors:** Gaurav Sharma, Zainab Khan, Deepanker Das, Surya Singh, Samradhi Singh, Manoj Kumar, R. R. Tiwari, Devojit Kumar Sarma

**Affiliations:** 1 ICMR-National Institute for Research in Environmental Health, Bhopal, Madhya Pradesh India; 2 ICMR-National Institute of Research in Tribal Health, Jabalpur, Madhya Pradesh India; 3 Somaiya Vidyavihar University, Mumbai, Maharashtra India

**Keywords:** Dengue, *Aedes aegypti*, Temperature, Biology, Climate change, Environment, Morphometrics

## Abstract

**Background:**

The geographic expansion of *Aedes aegypti*, an arboviral disease vector of global importance, is driven by urbanization, global travel, and climate change. Temperature significantly impacts the life cycle, distribution, and vectorial capacity of disease vectors. This study investigates the effects of temperature on the developmental biology, survival, reproductive traits, and wing morphometry of *Ae. aegypti* populations from central India (Bhopal, Madhya Pradesh).

**Methods:**

Larvae collected from the field were reared at controlled temperatures, on the basis of the historical and projected temperature changes, ranging from 10 ℃ to 40 ℃. *Aedes* stage-specific developmental times and survivorship rates were determined and compared. The right wings of male and female mosquitoes reared at 20 °C, 26 °C, and 32 °C were used for morphometric analysis on the basis of the digitized coordinates of 18 landmarks on the wing veins.

**Results:**

Higher temperature (32 °C) significantly accelerated life cycle completion, whereas 37 ℃ led to larval survival but high pupal mortality. In contrast, moderate temperatures (26 °C) optimized survival, reproductive output, and extended oviposition periods. Life table analysis revealed that elevated temperatures, particularly at 32 ℃, increased the intrinsic rate of population growth (*r*_m_) and shortened generation times, indicating faster population turnover under warmer conditions. However, this rapid life cycle presents trade-offs, including lower survival and reproductive success, which could significantly impact vector population dynamics in the context of climate-driven temperature fluctuations. Wing morphometric analysis further revealed that mosquitoes reared at 32 °C and 26 °C had significantly smaller wings compared with those reared at 20 °C. Although smaller wings may limit dispersal capacity, previous studies suggest a possible link with increased host-seeking and enhanced vectorial potential at 32 °C.

**Conclusions:**

This study highlights that *Ae. aegypti* populations from Central India exhibit thermal tolerance and developmental plasticity under elevated temperatures, suggesting their potential to thrive in warm climates. Rapid development and smaller wing size at higher temperatures may influence survival, fecundity, and biting behavior. Such traits can enhance disease transmission risks by supporting more frequent human–vector contact and sustaining mosquito populations in broader geographic areas.

**Graphical Abstract:**

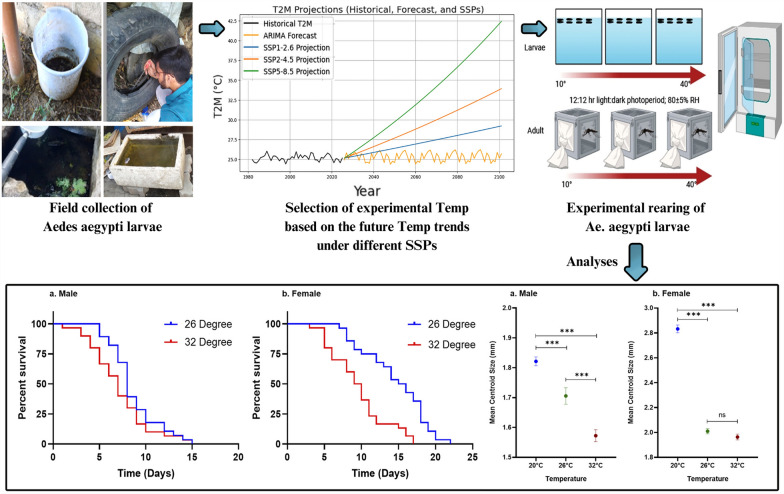

**Supplementary Information:**

The online version contains supplementary material available at 10.1186/s13071-025-06924-7.

## Background

Global climate change has significantly altered the geographical distribution of mosquito species, influencing the spread of many vector-borne diseases [1]. The range of *Aedes aegypti*, a key vector of diseases such as dengue, Zika, yellow fever, and chikungunya, has expanded across continents owing to these climatic shifts [2, 3]. This species likely originated in the tropical and subtropical regions of Africa and spread globally through human trade and migration, including the transatlantic slave trade. Its remarkable adaptability to urban environments, where it thrives in proximity to humans and utilizes artificial water containers for breeding, has made it a formidable vector species [2].

The global burden of dengue alone surpassed a record 14 million cases and 9000 deaths in 2024, more than doubling the historic milestone of 7 million cases observed in 2023 [4]. In India, *Ae. aegypti* has spread across all geo-climatic zones, contributing to approximately 250,000 dengue cases and 300 deaths in 2024 [5, 6]. Being an ectotherm, mosquito development, physiology, and behavior are directly influenced by environmental temperature [7] and thus are sensitive to both periodic and sustained changes in global and local climate [8], resulting in a differential capability of disease transmission. Therefore, understanding the population dynamics of immature *Ae. aegypti* and factors affecting their development is essential for managing *Aedes*-borne diseases.

Numerous studies highlight the effects of various physical and environmental factors, especially temperature, nutrition, and population density, on the development and survival of the *Aedes* mosquito [9–13]. The relationship between temperature and mosquito growth rates is critical for designing effective vector control models. Research shows that higher temperatures are associated with increased mortality, reduced adult body size, and lower oviposition rates in *Ae. aegypti* [14]. Mordecai et al. [15] demonstrated that transmission of dengue, Zika, and chikungunya viruses occurs between 18 °C and 34 °C, with peak transmission between 26 °C and 29 °C. However, above this optimal range, transmission decreases owing to thermal constraints on mosquito survival and viral replication. This indicates the importance of studying temperature-dependent mosquito traits to predict future transmission potential. In addition to temperature, larval nutrition plays a crucial role in mosquito development and fitness. Laboratory studies have shown that mosquitoes reared under resource-limited conditions exhibit prolonged development times, increased juvenile mortality, and reduced adult body size [16]. These resource constraints influence vector population dynamics, a factor often overlooked in climate-based disease transmission models. Moreover, Mackay et al. [17] found that larval nutrition significantly alters immune gene expression in adult mosquitoes, suggesting that environmental conditions during early development may impact adult immune susceptibility and transmission potential.

*Aedes aegypti* populations display a significant genetic and phenotypic variation in response to local climatic conditions, indicating possible local adaptation [18–21]. Understanding this adaptation is crucial for predicting how *Ae. aegypti* populations will respond to region-specific climatic trends and their impact on disease transmission. While a few studies have reported variations in life history traits among Western and Eastern Indian populations of *Ae. aegypti* [18], empirical data from the Central Indian region remain largely absent despite its distinct climatic conditions and frequent dengue outbreaks [22]. Recent modeling studies [23, 24] have forecasted the geographical expansion of *Ae. aegypti* under different climate change scenarios in different parts of India, including Central India. A 41.1% increase in the potential suitability of *Ae. aegypti* habitat was predicted for India under the Shared Socioeconomic Pathway 3 (SSP3) scenario, with annual mean temperature identified as a major contributing factor [24]. These findings highlight the importance of investigating the effect of different temperature conditions on the biology of *Aedes* mosquitoes in terms of potential climate change scenarios in this region. To address this, the present study provides the first empirical data on temperature-dependent biology and wing morphometry of *Ae. aegypti* from central India. Owing to the complexities of assessing temperature effects under natural conditions, the study was conducted in a controlled laboratory setting to simulate natural conditions and assess the effects of different temperature regimens on the developmental time, survival, life table parameters, and wing morphometrics of *Ae. aegypti* populations in central India.

## Methods

### Study site, mosquito collection, and colony development

The larvae and pupae of *Ae. aegypti* mosquitoes were collected from various larval habitats, including cement tanks and discarded tires, in urban areas of Bhopal city, Madhya Pradesh, Central India, during January 2023. The immature stages of *Ae. aegypti* were transported to the laboratory at the Indian Council of Medical Research - National Institute for Research in Environmental Health (ICMR-NIREH), Bhopal, where they were reared under controlled laboratory conditions at 26 ± 1 °C, 80 ± 5% relative humidity, and a 12:12-h light–dark photoperiod using an insect growth chamber (Percival Scientific, USA), as described previously [18]. The larvae were reared in plastic trays containing 1.5 L of autoclaved tap water and were fed a diet consisting of yeast extract and dog food powder (35.0 µg/L). Adult mosquitoes were provided with blood meals using a membrane feeding system modified from glass containers and maintained at 37 °C [25]. The goat blood used for feeding was sourced weekly from a certified slaughterhouse, ensuring it was collected from healthy animals. Efforts were made to ensure fresh blood availability each week for mosquito feeding. Consent was obtained from the slaughterhouse owners regarding the use of the blood for research purposes. Oviposition surfaces consisted of Whatman filter paper, folded into cylindrical shapes, and placed in water-filled cups. After the eggs were laid, the papers were kept moist for 2–3 days to facilitate embryonation, then dried for 24 h and stored in plastic containers. The laboratory-produced eggs were subsequently used as specimens for further experimental studies. 

### Calculation of future temperature projections for Bhopal city

**Fig. 1:**
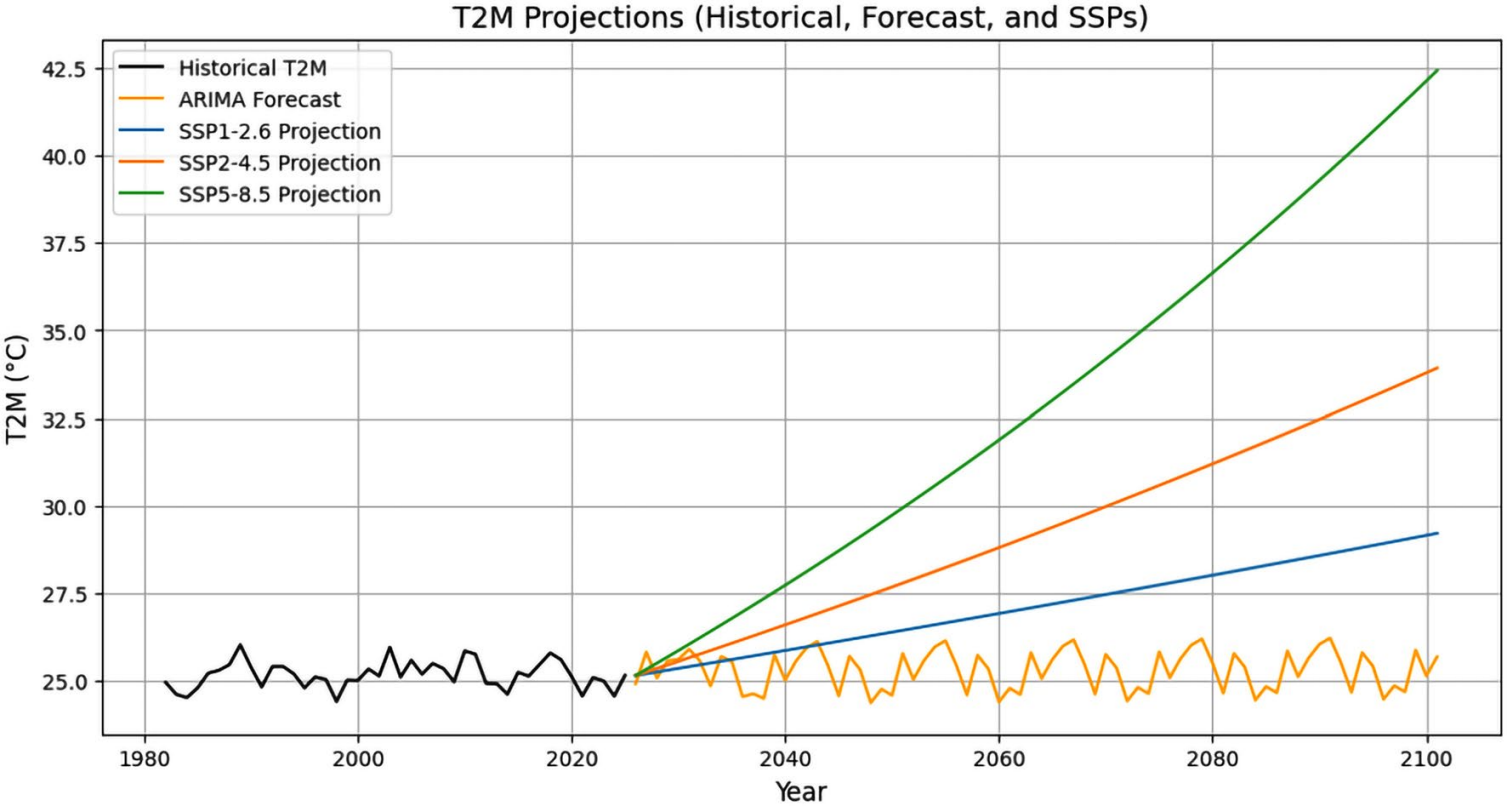
Time series of mean surface temperature (T2M) change of Bhopal city, Madhya Pradesh, Central India from 1981–2024 (black) with future forecast using ARIMA model (yellow) and future simulations for three SSP-Representative Concentration Pathways (RCPs) (SSP1–2.6 (blue), SSP2–4.5 (orange), and SSP5–8.5 (green)) from 2025 to 2100, respectively

To estimate the life history traits of *Ae. aegypti* under different temperature conditions relevant to present and future climate scenarios, we calculated temperature tendencies for Bhopal city using historical daily mean temperatures for the period 1981–2024. The temperature data were retrieved from the National Aeronautics and Space Administration (NASA) Langley Research Center Prediction of Worldwide Energy Resource Project (https://power.larc.nasa.gov/data-access-viewer). We projected these data in terms of different shared socioeconomic pathways (SSPs) to obtain an estimate of future temperature estimates using Python scripts. Details of the scripts are available in the Additional File 1: Supplementary Text S1. SSPs are used in climate modeling to explore different future scenarios of global development and their impact on climate change. These pathways generate different estimated temperatures by assuming varying levels of greenhouse gas (GHG) emissions, technological advancements, and policy efforts to mitigate climate change. Higher emissions (SSP5) lead to greater radiative forcing and warming, while lower emissions (SSP1) limit temperature rise [26]. On the basis of the existing data, the average mean temperature of Bhopal city was found to be 25.1 (± 0.39) ℃, and the projected mean temperature was observed to be 26.0 ℃, 26.5 ℃, and 27.8 ℃ (for the year 2040); 27.0 ℃, 28.5 ℃, and 32.0 ℃ (for the year 2060); 28.0 ℃, 31.0 ℃, and 36.7 ℃ (for the year 2080); and 28.7 ℃, 34.0 ℃, and 42.0 ℃ for the year 2100 under SSP1, SSP2, and SSP5 scenarios, respectively (Fig. 1). On the basis of this, we selected seven different temperature conditions, i.e., 10, 15, 20, 26, 32, 37, and 40 ℃, for the mosquito-rearing experiments.

### Estimation of development time and survival

Eggs from the F_3_ generation were used to estimate development time and survival rates across a range of temperature regimes, i.e., 10 ± 1 °C, 15 ± 1 °C, 20 ± 1 °C, 26 ± 1 °C, 32 ± 1 °C, 37 ± 1 °C, and 40 ± 1 °C. The conditions included a 12:12-h light–dark photoperiod and 80 ± 5% relative humidity. For each temperature regime, Whatman filter paper containing 50 eggs per replicate was used, with three replicates in total. Each cohort was placed in a 1.5 L water-filled tray, and the larvae were fed a mixture of yeast extract powder and a dog biscuit. Larvae in each cohort were counted daily and classified by instar stage, following the method described by Grech et al. [9]. Once larvae reached the pupal stage, they were removed from the trays and placed in 100 ml plastic cups covered with netting and containing 50 ml of water, where they were allowed to emerge as adults. The cohorts were monitored until complete adult emergence. Trays and cups were inspected daily, and water and food were changed every other day to maintain constant volumes. The number of days spent in each preimaginal stage was recorded for each cohort to estimate the development time for each larval instar and the pupal stage. The presence of exuviae indicated transitions between instar stages. The date of adult emergence was noted, and the total development time was calculated as the sum of days spent in each stage, from egg hatching to adult emergence. The immature survival percentage was calculated by tracking the number of larvae that successfully progressed to the next instar stage and completed pupation. 

### Estimation of adult survival and fecundity

To estimate the adult life table, two temperature regimes, 26 °C and 32 °C, were selected. These temperatures were chosen because the other regimes used in the developmental study did not produce enough adult mosquito pairs for life table analysis. For each temperature, three replicates were prepared, with each replicate containing ten male and ten female mosquitoes (1–2 days post-emergence), reared under a 12:12-h light–dark photoperiod and 80 ± 5% relative humidity. The adults were provided cotton pads soaked in a 10% sucrose solution for feeding. The selection of ten pairs of adults for the adult life table analysis was owing to mortality observed during development. Although 50 eggs per replicate were initially taken, only ten pairs of same-age adults survived to adulthood. To determine life expectancy (*e*_x_) for males and females at emergence and the mean survival time, daily mortality observations were recorded until all mosquitoes in each replicate died.

The paired mosquitoes were starved for 24 h and then blood-fed on the fifth day. They were then allowed 24 h to develop eggs. Following this, all batches of gravid females were given 150 ml containers lined with oviposition paper (30 cm in length, 15 cm in width) for egg-laying. Eggs from both the oviposition paper and the water surface were collected and counted daily under a microscope (WILD M3C microscope, Switzerland). To estimate fecundity-related parameters, data were recorded on the maximum oviposition days per cohort (the number of days between the first and last oviposition event during the lifespan), eggs per female per day (the average number of eggs laid by a female alive on a given day), eggs per female over the lifespan, and total egg production per cohort of 20 females, following the method described by Sharma et al. [18].

### Estimation of adult life table attributes

The life table attributes, such as net reproductive rate (*R*_0_), intrinsic rate of increase (*r*_m_), finite rate of increase (*λ*), doubling time (DT), and mean generation time (*T*) were estimated as per the methods of Sharma et al. [18].

### Estimation of wing morphometrics

**Fig. 2:**
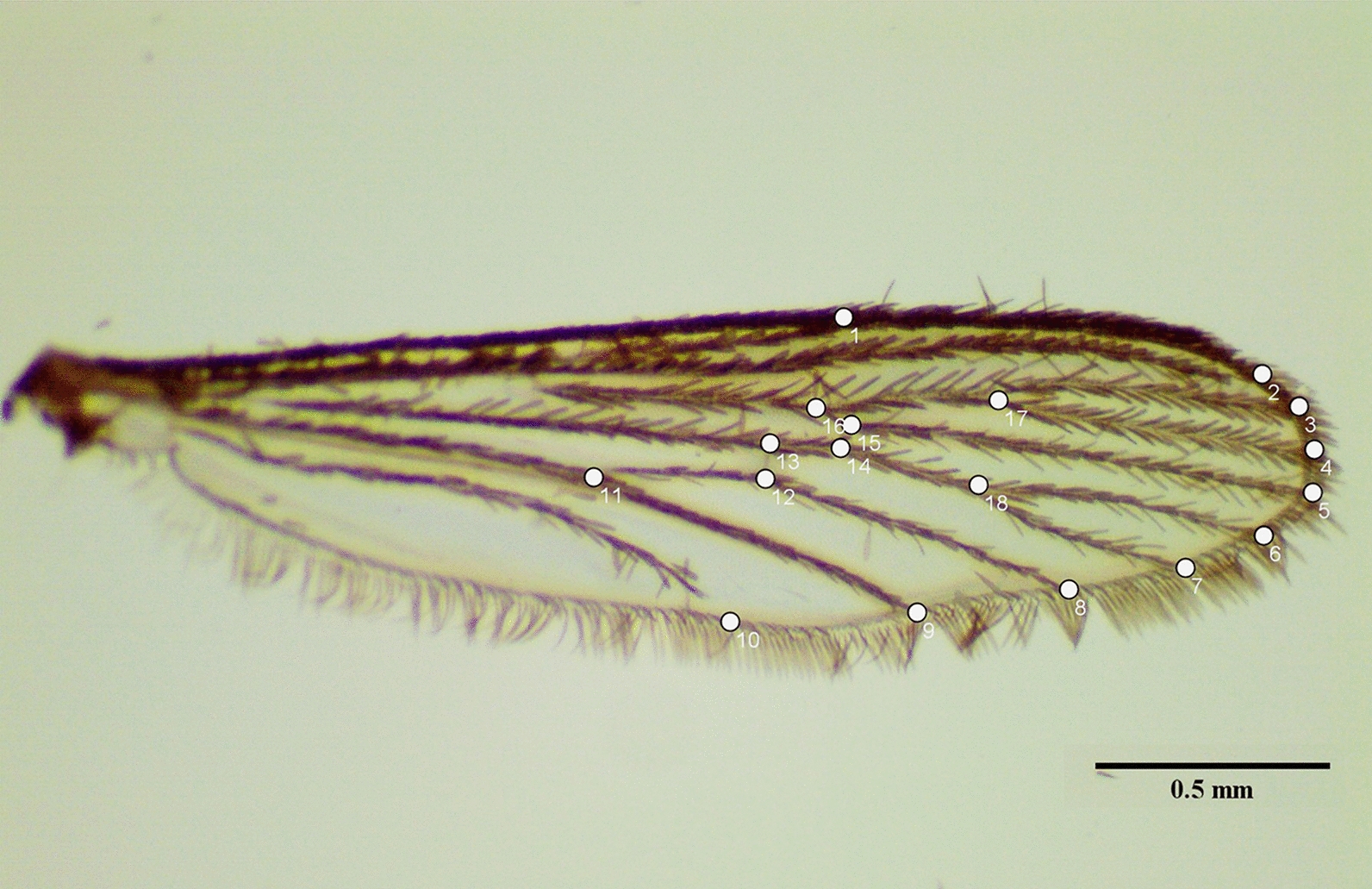
Representation of the 18 landmarks digitized on wings of *Ae. aegypti* using ImageJ software

To analyze the effect of temperature on wing morphometrics, three temperature regimes: 20 °C, 26 °C, and 32 °C were selected, as other conditions did not yield sufficient adult mosquito specimens. A total of 161 wing specimens from both male and female *Ae. aegypti* mosquitoes were examined, comprising 25, 23, and 16 males, and 33, 38, and 26 females, respectively, for the 20 °C, 26 °C, and 32 °C temperature conditions. The right wing of each male and female mosquito was dissected from the thorax and mounted on microscope slides (15 mm × 15 mm) with DPX (Dibutylphthalate Polystyrene Xylene) and a coverslip. The wings were then photographed using ImageView software (version ×64, 110 4.11.18012.20201123) with a STEMI 305 stereo microscope at 40× magnification. On the basis of these photographs, a total of 18 landmarks on each wing were digitized using ImageJ software (version 1.54d), following the methods outlined by Hounkanrin et al. [19]. The distribution of the 18 landmarks is shown in Fig. 2.

### Statistical analysis

#### Immature development time and survival

To assess the development time and survival rates of the larval and pupal stages of *Ae. aegypti* reared under different temperature conditions, a multiple sample comparison test was performed using one-way analysis of variance (ANOVA), followed by Bonferroni post hoc correction, in the PASW version 18.0 statistical software package.

#### Life table attributes

Adult mean survival time was estimated using the log-rank (Mantel–Cox) test. Various life table parameters, such as *R*_0_, *r*_m_, *λ*, DT, and *T* of 26 °C- and 32 °C-reared strains were subjected to a *t*-test at *P* < 0.05. For all the statistical analyses, the PASW version 18.0 statistics software package was used.

#### Wing size and shape

The centroid size (CS) was considered as the wing size [27]. Coordinates were imported into the MorphoJ version 1.08.01 [28] in text-delimited format. These coordinates were aligned by performing Procrustes superimposition to visualize the position of each landmark for each specimen using MorphoJ version 1.08.01 [28]. To statistically compare the mean CS among specimens from different temperature variations, one-way ANOVA followed by Tukey’s honestly significant difference (HSD) test for multiple comparisons of means was performed using GraphPad prism version 8.0. The assessment of wing shape involved the importation of wing coordinates in a text-delimited format using MorphoJ version 1.08.01 [28]. To conduct a comparative analysis, Procrustes ANOVA was performed utilizing the same software. Following this, canonical variate analysis (CVA) (70% confidence) was applied to visually represent shape variations on the basis of Procrustes coordinates.

## Results

### Immature development time and survival

The developmental durations of the various stages, i.e., 1st instar (L1), 2nd instar (L2), 3rd instar (L3), 4th instar (L4), and pupal (P) stage of *Ae. aegypti* were significantly influenced by temperature. The longest durations were consistently observed at 20 °C across all stages (L1, 2.7 ± 0.2 days; L2, 1.9 ± 0.2 days; L3, 2.2 ± 0.1 days; L4, 4.4 ± 0.3 days; P, 3.5 ± 0.1 days). In contrast, the shortest durations were generally found at higher temperatures, particularly at 32 °C (L1, 1.1 ± 0.1 days; L2, 1.0 ± 0.0 days; L3, 1.0 ± 0.0 days) and 37 °C (L4, 1.4 ± 0.2 days; P, 1.0 ± 0.0 days) (Table 1). Similarly, the highest survival rates were observed at 26 °C (L4, 100.0 ± 0.0%; P, 99.1 ± 0.9%) and 32 °C (L1, 91.3 ± 8.7%; L2, 99.3 ± 0.7%; L3, 100.0 ± 0.0%), while the lowest survival rates were recorded at 37 °C (L1, 28.0 ± 2.3%; L2, 66.0 ± 10.4%; L3, 75.6 ± 13.4%; L4, 59.7 ± 24.1%; P, 0.0 ± 0.0%) (Table 2).

**Table 1:** Comparison of development time (days) of larval and pupal stages of *Ae. aegypti* reared under four different temperature conditions using ANOVA (mean ± standard error (SE))

Stage	20 °C	26 °C	32 °C	37 °C	*P*-value
L1	2.7 ± 0.2^a^	1.6 ± 0.1^b^	1.1 ± 0.1^c^	1.1 ± 0.0^cd^	0.00^*^
L2	1.9 ± 0.2^a^	1.0 ± 0.0^b^	1.0 ± 0.0^b^	1.3 ± 0.3^ab^	0.03^*^
L3	2.2 ± 0.1^a^	1.1 ± 0.1^b^	1.0 ± 0.0^b^	1.1 ± 0.9^b^	0.00^*^
L4	4.4 ± 0.3^a^	1.7 ± 0.2^b^	1.9 ± 0.2^b^	1.4 ± 0.2^b^	0.0001^*^
P	3.5 ± 0.1^a^	1.9 ± 0.1^b^	1.6 ± 0.0^c^	1.0 ± 0.0^d^	0.00^*^
Total larva	11.2 ± 0.3^a^	5.4 ± 0.2^b^	5.0 ± 0.2^b^	4.9 ± 0.4^b^	0.70
L + P	14.7 ± 0.3^a^	7.4 ± 0.1^b^	6.6 ± 0.2^bc^	5.9 ± 0.4^c^	0.00^*^

^a,b,c,d^Mean values within the same row with different letters are significantly different (*P* < 0.05); L1, 1st instar; L2, 2nd instar; L3, 3rd instar; L4, 4th instar; P, pupa

^*^Statistically significant

**Table 2:** Comparison of survival % of larval and pupal stages of *Ae. aegypti* reared under four different temperature conditions using ANOVA (mean ± SE)

Stage	20 °C	26 °C	32 °C	37 °C	*P*-value
L1	64.7 ± 17.0^a^	82.0 ± 4.2^a^	91.3 ± 8.7^a^	28.0 ± 2.3^b^	0.0084^*^
L2	93.2 ± 1.0^a^	96.5 ± 2.2^a^	99.3 ± 0.7^a^	66.1 ± 10.4^b^	0.0082^*^
L3	88.7 ± 4.5^ab^	99.1 ± 0.9^a^	100.0 ± 0.0^a^	75.6 ± 13.4^b^	0.0959
L4	96.6 ± 1.7^ab^	100.0 ± 0.0^a^	97.3 ± 1.3^ab^	59.7 ± 24.1^b^	0.1321
P	79.4 ± 9.2^a^	99.1 ± 0.9^b^	92.6 ± 1.6^ab^	0.0 ± 0.0	0.0484^*^
Total larva	51.3 ± 13.4^a^	78.7 ± 4.8^b^	88.0 ± 7.0^b^	8.0 ± 3.1^c^	0.0005^*^
L + P	42.0 ± 13.6^a^	78.0 ± 5.0^b^	81.3 ± 5.9^b^	0.0 ± 0.0	0.0395^*^

^a,b,c,d^Mean values within the same row with different letters are significantly different (*P* < 0.05); L1, 1st instar; L2, 2nd instar; L3, 3rd instar; L4, 4th instar; P, pupa

^*^Statistically significant

### Adult survival

**Fig. 3:**
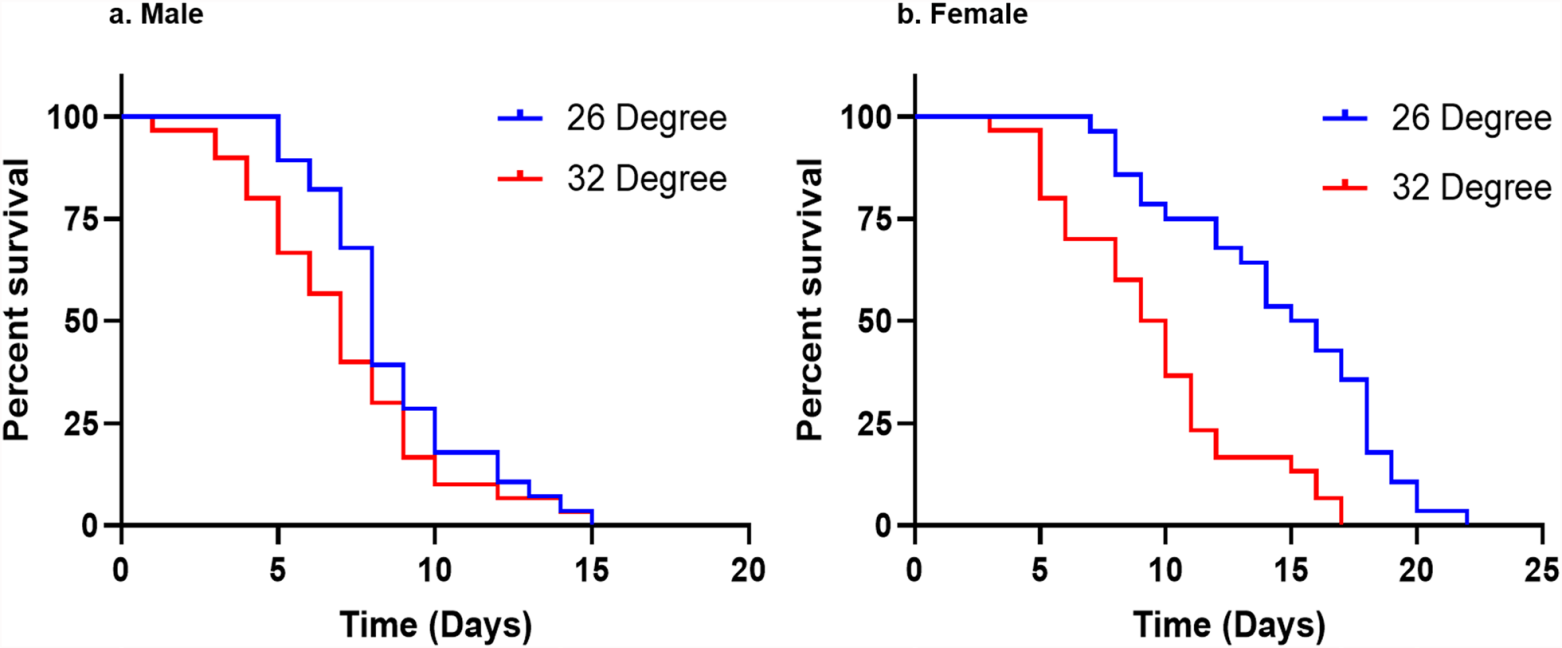
Kaplan–Meier survival plot of *Ae. aegypti* showing the developmental dynamics in two different temperature conditions. (**a**) Male mosquito; (**b**) female mosquito (sample sizes for male and female mosquitoes are 10, 10, and 8 for each replicate and each temperature regime)

**Fig. 4:**
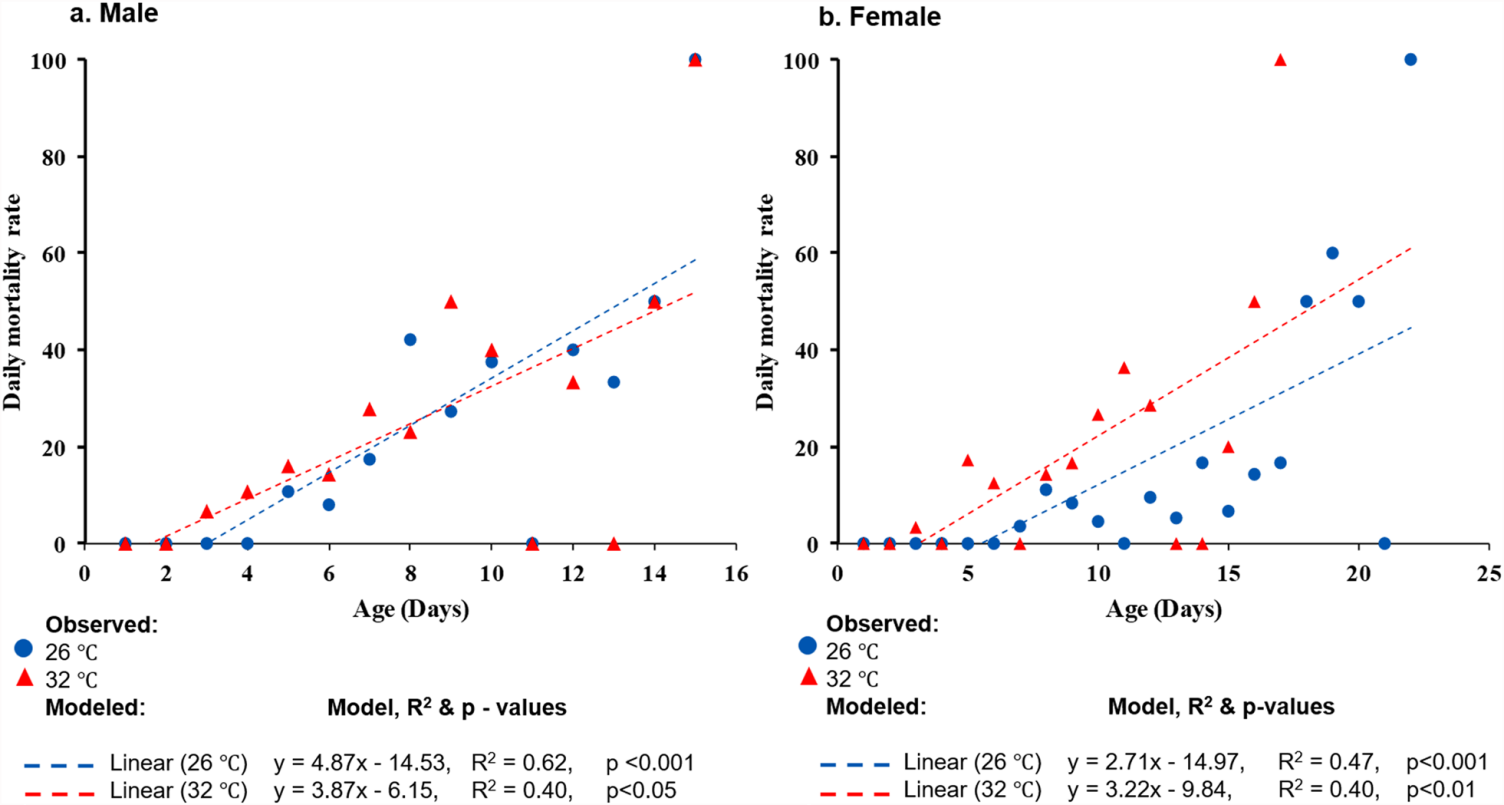
Age-specific daily mortality rate of *Aedes aegypti* in the two tested temperature conditions. (**a**) Male mosquito; (**b**) female mosquito (sample sizes for male and female mosquitoes are 10, 10, and 8 for each replicate and each temperature regime)

Log rank (Mantel–Cox) survival analysis revealed that the mean survival time for female mosquitoes was longer at 26 °C (14.6 ± 0.8 days) compared with 32 °C (9.5 ± 0.7 days) (Fig. 3). For males, the mean survival time was recorded as 9.2 ± 0.9 days at 26 °C and 12.0 ± 1.7 days at 32 °C (Fig. 3). However, a significant difference was observed only in female survival (*χ*^2^ = 18.6, *df* = 1, *P* = 0.0). Age-dependent mortality was observed in both male and female adult mosquitoes at both temperatures. Significant positive slope values in the model indicated that older mosquitoes experienced higher mortality rates (Fig. 4). However, no significant difference in the age-related mortality rate was observed between the two temperature conditions either in males (*t*_(28)_ = 0.0369, *P* = 0.97) or in females (*t*_(34.7)_ = 0.3552, *P* = 0.72). In terms of life expectancy (*e*_x_), both males and females lived longer at 26 °C, with males averaging 3.3 ± 0.1 days and females 4.7 ± 1.5 days. At 32 °C, the life expectancy of males was 3.2 ± 0.5 days, while that for females was 4.2 ± 0.6 days (Table 3). However, the difference in life expectancy between males and females was not statistically significant.

**Table 3:** Comparison of various adult life table attributes of *Ae. aegypti* populations reared under two different temperature conditions using *t*-test (mean ± SE)

Statistical Parameters	26 °C	32 °C	*P*-value
Maximum male survival (days)	9.2 ± 0.9	12.0 ± 1.7	0.15
Maximum female survival (days)	14.6 ± 0.8	9.5 ± 0.7	0.001^*^
Male life expectancy (*e*_x_) (at emergence; days)	3.3 ± 0.1	3.2 ± 0.5	0.66
Female life expectancy (*e*_x_) (at emergence; days)	4.7 ± 1.5	4.2 ± 0.6	0.73
Eggs/female/day	14.6 ± 5.9	16.6 ± 10.6	0.75
Eggs/female lifespan	101.5 ± 5.5	70.7 ± 9.9	0.47
Total egg production/cohort of 10 females	516.7 ± 31.9	255.0 ± 37.8	0.051
Maximum oviposition days/cohort	12.3 ± 0.9	7.0 ± 2.0	0.07
Intrinsic rate of increase (*r*_m_)	0.2 ± 0.0	0.3 ± 0.0	0.87
Finite rate of increase (*λ*)	1.27 ± 0.1	1.29 ± 0.0	0.90
Mean generation time (*T*)	13.9 ± 2.1	10.1 ± 1	0.17
Doubling time (DT)	3.0 ± 0.6	2.7 ± 0.2	0.69
Net reproductive rate (*R*_0_)	25.8 ± 4.3	13.3 ± 1.6	0.03^*^

^*^Statistically significant

### Fecundity

Mosquitoes reared at 32 °C laid a higher number of eggs per female per day (16.6 ± 10.6) compared with those reared at 26 °C (14.6 ± 5.9). However, other fecundity-related traits, such as total eggs per female lifespan, total egg production per cohort of ten females, and net reproductive rate (*R*_0_), were higher at 26 °C. Specifically, at 26 °C, the total number of eggs per female over the lifespan was 101.5 ± 5.5, total egg production per cohort was 516.7 ± 31.9, and the net reproductive rate was 25.8 ± 4.3. In contrast, at 32 °C, these values were lower: 70.7 ± 9.9 eggs per female lifespan, 255.0 ± 37.8 total egg production per cohort, and a net reproductive rate of 13.3 ± 1.6. A statistically significant difference was observed only in the net reproductive rate between the two temperatures (*P* = 0.03, Table 3).

### Oviposition schedule

The duration of oviposition was longer at 26 °C, with an average of 12.3 ± 0.9 days per cohort, compared with 7.0 ± 2.0 days at 32 °C. However, this difference was not statistically significant (*P* = 0.25).

### Adult life table attributes

Mosquitoes reared at 32 °C exhibited a slightly higher intrinsic rate of increase (*r*_m_, 0.3 ± 0.0 days) and finite rate of increase (*λ*, 1.29 ± 0.0 days) compared with those reared at 26 °C (*r*_m_, 0.2 ± 0.0 days; *λ*, 1.27 ± 0.0 days), though these differences were not statistically significant (Table 3). Conversely, the mean generation time (*T*) and doubling time (DT) were longer at 26 °C, averaging 13.9 ± 2.1 days and 3.0 ± 0.6 days, respectively (Table 3).

### Wing morphometry

**Fig. 5:**
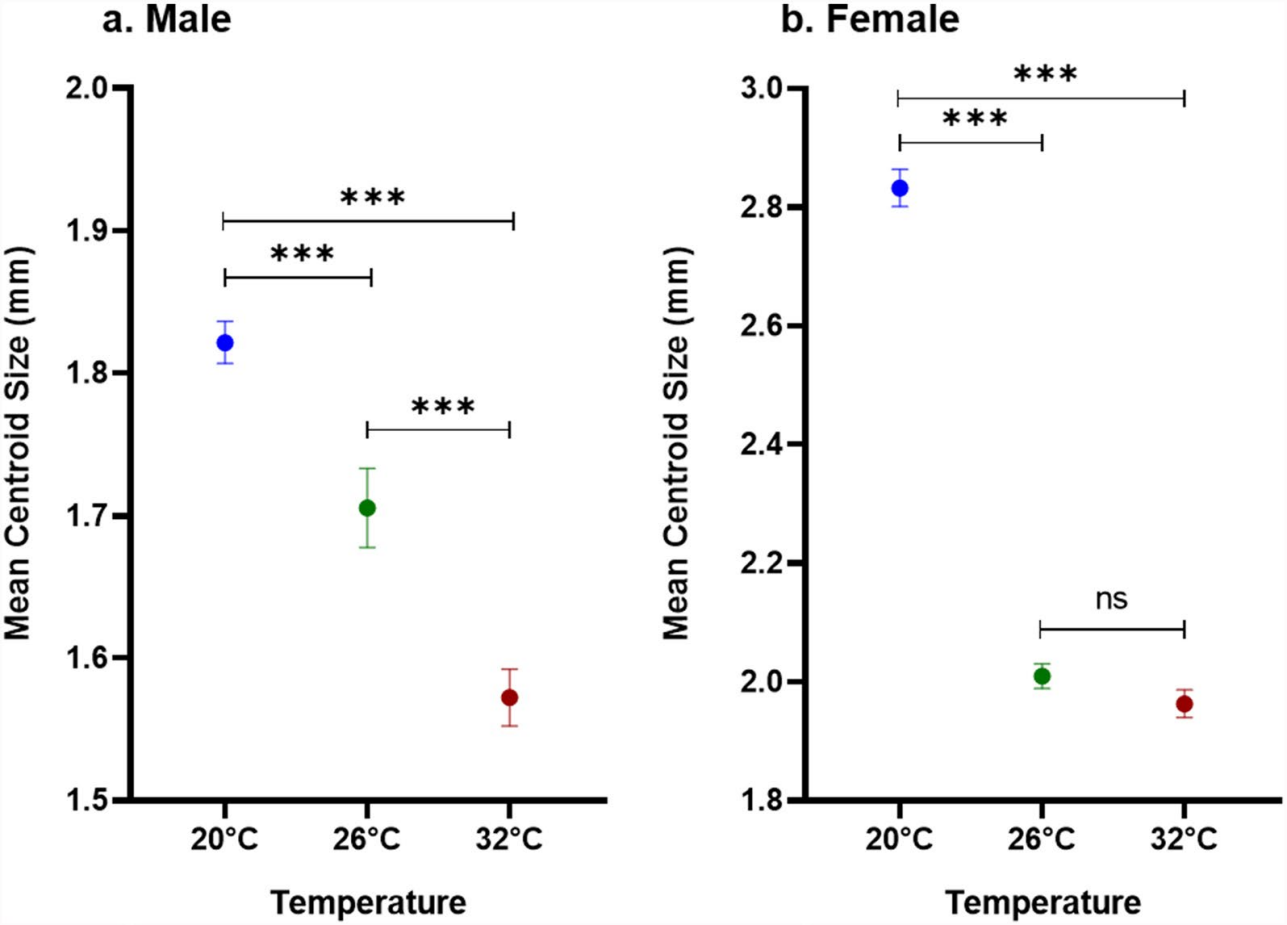
Variation in wing size of *Ae. aegypti* in response to different temperature conditions. (**a**) Male mosquito; (**b**) female mosquito (sample sizes were 25, 23, and 16 for males and 33, 38, and 26 for females, respectively, for the 20 °C, 26 °C, and 32 °C temperature conditions. Statistical significance was determined by one-way ANOVA followed by Tukey’s multiple comparison. Error bars represent the standard error of the mean; ^***^Represents significance at level ≤ 0.001)

**Fig. 6:**
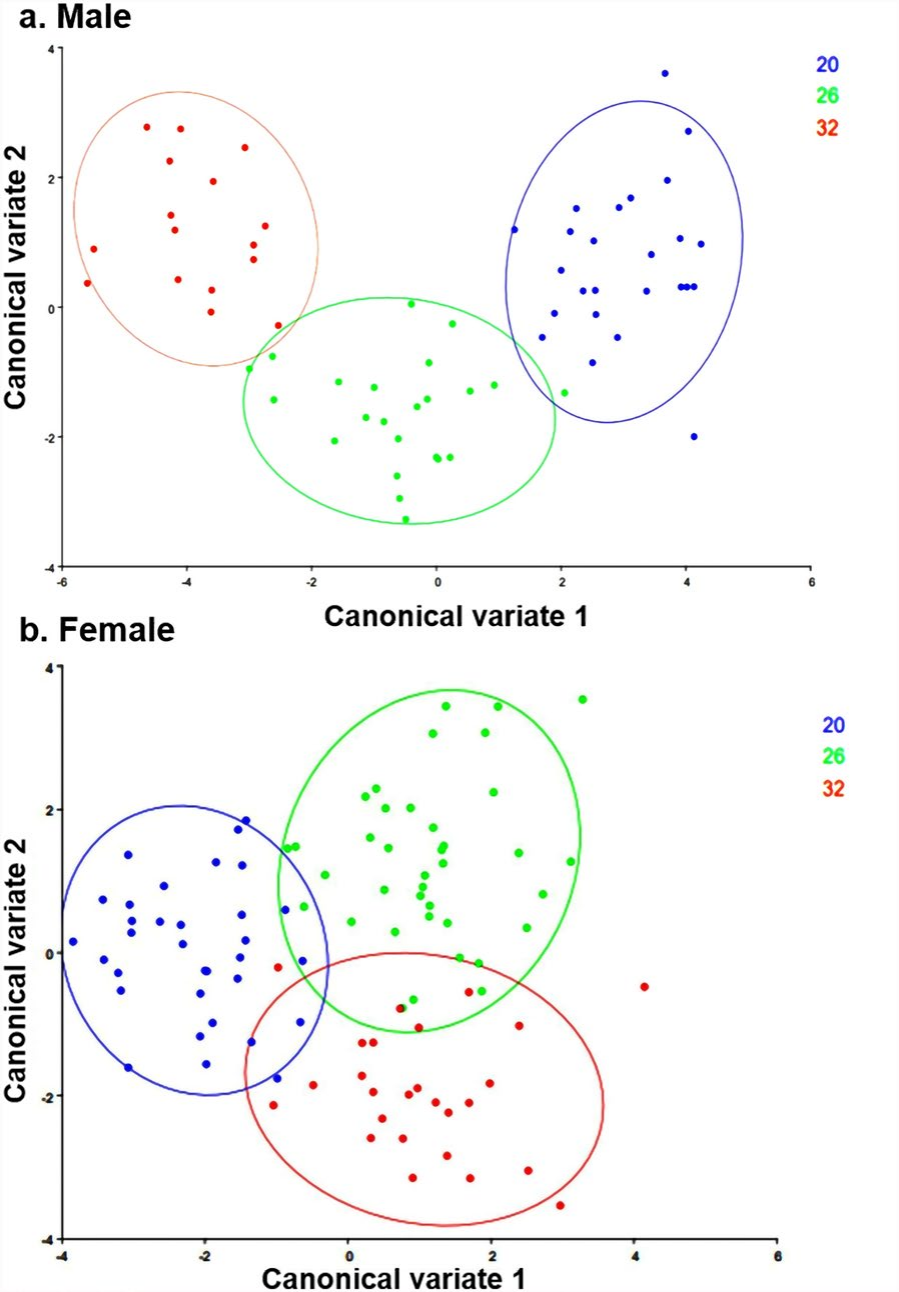
Canonical variate analysis of the Procrustes coordinates of *Aedes* *aegypti* mosquitoes. (**a**) Male mosquito; (**b**) Female mosquito (sample sizes were 25, 23, and 16 for males and 33, 38, and 26 for females, respectively, for the 20 °C, 26 °C, and 32 °C temperature conditions)

The present study found a significant impact of temperature on wing morphometry. In terms of wing size, the wings were larger at 20 °C (male, 1.8 ± 0.0 mm; female, 2.8 ± 0.0 mm), while at 32 °C, the wing size was smaller (male, 1.6 ± 0.0 mm; female, 1.9 ± 0.0 mm). These differences were highly significant (*P* < 0.0001) (Fig. 5). The analysis of wing shape variation using Procrustes ANOVA also showed significant differences across the temperature conditions (male, *F* ratio = 3.29, *P* < 0.0001; female, *F* ratio = 6.94, *P* < 0.0001). Canonical variate analysis (CVA) explained 99.9% of the total variance among the temperature conditions (male, CV1 = 83.4%, CV2 = 16.6%; female, CV1 = 59.6%, CV2 = 40.4%). The scatter plots of CV1 and CV2 for males and females demonstrated clear separation, highlighting distinct wing shape variation (Fig. 6).

## Discussion

The present study examined the impact of temperature on the developmental stages, survival rates, adult life table traits, and wing morphometrics of *Ae. aegypti* populations, considering future rise in temperature under different climate change projections for Bhopal, Madhya Pradesh, India. The findings offer valuable insights into how temperature influences the biological parameters and fitness of this mosquito species, which is a primary vector of various arboviral diseases.

It was observed that temperature plays a crucial role in the developmental time of *Ae. aegypti* across different larval and pupal stages. Our study revealed that the developmental duration of *Ae. aegypti* was longest at a lower temperature (20 ℃) and significantly shortened at higher temperatures, particularly at 32 ℃ for early larval developmental stages (L1–L3) and 37 ℃ for later stages (L4 and pupae). This pattern aligns with the findings from the Córdoba area of Argentina, a humid subtropical region, where the total developmental time of *Ae. aegypti* decreased from 21.9 days at 15.2 °C to 8.6 days at 25.3 °C [9]. Similarly, Mohammed et al. [14] observed early pupation by day 4 at 30–35 °C in Trinidad, further supporting the trend that warmer conditions accelerate *Ae. aegypti* development. In contrast to *Ae. aegypti*, a comparable thermal response was also reported in *Ae. albopictus* from Italy [29], where the shortest immature development period (8.8 days) occurred at 30 °C, while the longest (35 days) was recorded at 15 °C. These findings collectively indicate that elevated temperatures accelerate the life cycle of *Aedes* mosquitoes, potentially increasing their population densities. This phenomenon aligns with the projected impacts of climate change, including rising global temperatures and more frequent heat waves. Such changes are particularly relevant for subtropical regions (Central India), where these climatic shifts could amplify mosquito breeding and survival. In addition, global warming may render previously unsuitable cooler areas habitable for *Ae. aegypti*, facilitating geographic expansion and complicating mosquito control efforts [30].

However, survival rates were highest at 26 °C and 32 °C, particularly during the L1, L2, and L3 stages, but declined sharply at 37 °C (Table 2). This decline at extreme temperatures suggests that while higher temperatures accelerate development, they may also impose physiological stress on larvae, reducing overall survival [14, 31, 32]. A similar trend was observed in *Ae. aegypti* populations from Argentina [9], where immature survival increased with temperature, rising from 26% at 15.2 °C to 92% at 21.6 °C*.* However, unlike our study, their findings showed 57% survival at 25.3 °C, with no significant effect, indicating possible region-specific thermal tolerance. Similar temperature-dependent trends have also been observed in other *Aedes* species. For instance, in *Ae. albopictus* from Italy [29], immature survival was 50% at 15 °C and increased to 76% at 25 °C. However, unlike *Ae. aegypti*, survival declined at 30 °C (68%), indicating a lower thermal tolerance threshold at higher temperatures. Furthermore, Mohammed et al. [14] reported a drastic reduction in egg-hatching success (1.6%) at 34–35 °C, reinforcing our findings that elevated temperature negatively impacts immature survival. This dual effect suggests that elevated temperature may have region-specific consequences for *Ae. aegypti* populations. Moderate warming could boost populations in some areas, while extreme heat may suppress mosquito fitness in others.

Most adult life table parameters of *Ae. aegypti* reared under 26 °C and 32 °C were observed to be not significant, except for female survival and net reproductive rate (*R*_0_) (Table 3). The highest survival rate of adult *Ae. aegypti* was observed at 26 °C compared with 32 °C, whereas higher temperatures resulted in shorter lifespans. This aligns with findings from previous studies that reported the greatest longevity for *Ae. aegypti* males (27–29 days) and females (25–34 days) at 22 °C, with a significant decline at 33 °C (males, 14–23 days; females, 15–22 days) [10]. A similar trend was observed in *Ae. albopictus*, where the highest survival (64 days) was recorded at 25 °C, with considerable reductions at 10 °C, 15 °C, and 30 °C [29]. These findings indicate that moderate temperatures around 26 °C are optimal for *Ae. aegypti* survival, consistent with previous research emphasizing the role of temperature in shaping mosquito longevity and reproductive fitness [33, 34]. While *Ae. aegypti* demonstrates a strong association with tropical and subtropical climates, *Ae. albopictus* exhibits a broader thermal tolerance, particularly at lower temperatures (10 °C) [32]. This enhanced cold tolerance supports the ability of *Ae. albopictus* to establish populations in temperate regions, extending its geographic range. However, despite its longer lifespan under cooler conditions, *Ae. aegypti* appears to be more resilient to fluctuating environmental temperatures, which may contribute to its dominance in tropical environments.

Temperature also plays a crucial role in shaping mosquito fecundity. Females reared at 32 °C laid more eggs per day, while overall reproductive success, measured as total eggs per female lifespan and net reproductive rate, was highest at 26 °C. The decline in the net reproductive rate at higher temperatures highlights the trade-off between quantity and quality of reproduction, where higher temperatures may increase egg-laying frequency but decrease overall reproductive success over the mosquito’s lifespan. For example, *Ae. albopictus* population from China reared at moderate temperature conditions (23–28 °C) laid more eggs and had greater survival compared with those reared at elevated temperatures (31 °C) [35]. Likewise, *Ae. albopictus* population from Italy showed maximum fecundity at 25 °C, with significant declines observed at both lower and higher temperature extremes [29]. These observations suggest that areas experiencing moderate warming under climate change may reach optimal temperature thresholds, enhancing *Ae. aegypti* longevity and reproduction. Conversely, extreme heat may reduce fitness, although temporary population surges might occur during heatwaves due to faster reproductive cycles [7, 36, 37].

In addition to biological and life table attributes, wing morphometric analysis is increasingly recognized as a critical tool in designing effective vector management strategies. The present study explored the effect of temperature on the wing morphometrics of *Ae. aegypti* populations, reared at 20 °C, 26 °C, and 32 °C. The results demonstrated that temperature had a profound impact on both wing size and shape. Mosquitoes reared at 20 °C developed significantly larger wings compared with those reared at 32 °C. This reduction in wing size at higher temperatures has critical implications in the context of climate change. Rising global temperatures may lead to mosquito populations with smaller body sizes which can feed on blood more frequently compared with the larger individuals [38], thereby increasing human–mosquito contact and disease transmission. These physiological changes could impact their survival and dispersal potential, ultimately influencing the dynamics of disease transmission. Conversely, larger mosquitoes, with larger wings, tend to exhibit enhanced blood-feeding capacity, improved flight performance, and distinct metabolic profiles, which can enhance their vectorial capacity [39]. Moreover, larger mosquitoes may be less susceptible to viral infections, such as dengue or Sindbis, which could alter virus transmission dynamics within populations [40, 41]. Morphometric studies across different geo-climatic regions have also shown that wing size and shape variations are directly linked to local environmental conditions. For example, Hounkanrin et al. [19] reported significant morphometric variation in *Ae. aegypti* populations from three different landscape types (sylvatic, urban, and semi-urban). Similarly, in India, Sharma et al. [42] reported that variations in wing morphometrics of *Ae. aegypti* are influenced by different geo-climatic regions of India, which can, in turn, inform region-specific control measures. Although there is an ongoing debate on the impact of wing size on vector capacity, the present findings highlight the importance of integrating morphometric data into vector control strategies, as understanding how environmental factors influence mosquito morphology can guide interventions aimed at limiting mosquito dispersal and survival. Further experimental studies on *Ae. aegypti* artificially infected with dengue virus and reared in different temperature conditions may provide important insights into the role of wing sizes on disease transmission.

This study strongly suggests that future temperature conditions under the climate change scenario in Central India will facilitate the growth, expansion, and distribution of the *Ae. aegypti* population. With an annual average temperature of approximately 26 °C, the region already provides favorable conditions for *Ae. aegypti* development. As the primary vector of the dengue virus in the area [43], this species poses a sustained risk of dengue transmission. If the projected annual average temperature rises above the current average due to climate change, the population’s capacity to transmit dengue is expected to remain unchanged. In addition, warmer temperatures will promote faster life cycle completion and reproduction, leading to larger population sizes and smaller individuals, which can potentially alter the dynamics of dengue transmission in the studied region [40, 44].

Our study has certain limitations. Laboratory experiments were conducted under constant temperature conditions, which may not fully reflect the fluctuating environmental conditions experienced in natural settings. In addition, life table parameters could not be analyzed at 37 °C owing to complete pupal mortality, limiting our ability to generalize population responses at extreme temperatures. The study was also geographically limited to a single population from Bhopal, Central India, which has a humid subtropical climate (Cwa). To gain a more comprehensive understanding, future research should include *Ae. aegypti* populations from diverse climatic regions and examine their responses under varying temperature regimes. Despite these limitations, our findings revealed significant differences in both immature and adult life table traits across temperature conditions, supported by notable variations in wing morphometry. 

## Conclusions

The present study provides critical insights into how temperature influences the development, survival, reproduction, and wing morphometrics of *Ae. aegypti* populations from Central India. Our findings demonstrate that local populations of *Ae. aegypti* can complete their life cycle at elevated temperatures (32 °C), indicating thermal tolerance and potential local adaptation. The projected future climatic conditions in Central India are expected to increase the persistence of *Ae. aegypti* in the region, with clear implications for disease transmission. This study is among the first to generate region-specific biological data for *Ae. aegypti* in Central India, thus filling an important research gap. Temperature-induced changes in wing morphometrics further reflect the physiological plasticity of this vector, which may influence its dispersal ability and vectorial capacity. These findings reinforce the importance of integrating ecological, biological, and morphological data into vector surveillance and control strategies. Understanding such local adaptation is essential for forecasting future transmission dynamics and implementing more targeted and effective mosquito control strategies under changing climate conditions in dengue endemic regions.

## Supplementary Information

Additional File 1: Supplementary Text S1. Codes for calculation of future temperature projections.

## Data Availability

The dataset for this study is publicly available at https://zenodo.org/ under the the DOI https://doi.org/10.5281/zenodo.15728008.

## Abbreviations

*χ*^2^: Chi-squared
*df*: Degrees of freedom
*e*_x_: Life expectancy
*r*_m_: Intrinsic rate of increase
*λ*: Finite rate of increase
*T*: Mean generation time
DT: Dobling time
*R*_0_: Net reproductive rate
CVA: Canonical variate analysis

## References

[1] Thomson MC, Stanberry LR. Climate change and vectorborne diseases. N Engl J Med. 2022;387:1969–78. 10.1056/NEJMra2200092. 36416768 10.1056/NEJMra2200092

[2] Powell JR, Gloria-Soria A, Kotsakiozi P. Recent history of *Aedes aegypti*: vector genomics and epidemiology records. Bioscience. 2018;68:854–60. 30464351 10.1093/biosci/biy119 PMC6238964

[3] Kaye AR, Obolski U, Sun L, Hart WS, Hurrell JW, Tildesley MJ, et al. The impact of natural climate variability on the global distribution of *Aedes aegypti*: a mathematical modelling study. Lancet Planetary Health. 2024;8:e1079–87. 39674197 10.1016/S2542-5196(24)00238-9 PMC7617884

[4] Haider N, Hasan MN, Onyango J, Billah M, Khan S, Papakonstantinou D, et al. Global dengue epidemic worsens with record 14 million cases and 9,000 deaths reported in 2024. Int J Infect Dis. 2025. 10.1016/j.ijid.2025.107940. 40449873 10.1016/j.ijid.2025.107940

[5] Baruah K, Katewa A, Singh G, Dhingra N. Epidemiological stratification of dengue in India and strategic challenges. Dengue Bull. 2020;41:149–65.

[6] NCVBDC: dengue situation in India. https://ncvbdc.mohfw.gov.in/index4.php?lang=1&level=0&linkid=431&lid=3715 (2025). Accessed 02 Jun 2025.

[7] Reinhold JM, Lazzari CR, Lahondere C. Effects of the environmental temperature on *Aedes aegypti* and *Aedes albopictus* mosquitoes: a review. Insects. 2018. 10.3390/insects9040158. 30404142 10.3390/insects9040158 PMC6316560

[8] Thai KT, Anders KL. The role of climate variability and change in the transmission dynamics and geographic distribution of dengue. Exp Biol Med. 2011;236:944–54. 10.1258/ebm.2011.010402. 10.1258/ebm.2011.010402 21737578

[9] Grech MG, Sartor PD, Almiron WR, Luduena-Almeida FF. Effect of temperature on life history traits during immature development of *Aedes aegypti* and *Culex quinquefasciatus* (Diptera: Culicidae) from Cordoba city. Argentina Acta Trop. 2015;146:1–6. 10.1016/j.actatropica.2015.02.010. 25733491 10.1016/j.actatropica.2015.02.010

[10] Marinho RA, Beserra EB, Bezerra-Gusmao MA, Porto Vde S, Olinda RA, Dos Santos CA. Effects of temperature on the life cycle, expansion, and dispersion of *Aedes aegypti* (Diptera: Culicidae) in three cities in Paraiba. Brazil J Vector Ecol. 2016;41:1–10. 10.1111/jvec.12187. 27232118 10.1111/jvec.12187

[11] Cui G, Zhong S, Zheng T, Li Z, Zhang X, Li C, et al. Aedes albopictus life table: environment, food, and age dependence survivorship and reproduction in a tropical area. Parasit Vectors. 2021;14:568. 10.1186/s13071-021-05081-x. 34743753 10.1186/s13071-021-05081-x PMC8573987

[12] Delatte H, Gimonneau G, Triboire A, Fontenille D. Influence of temperature on immature development, survival, longevity, fecundity, and gonotrophic cycles of *Aedes albopictus*, vector of chikungunya and dengue in the Indian Ocean. J Med Entomol. 2009;46:33–41. 10.1603/033.046.0105. 19198515 10.1603/033.046.0105

[13] Brady OJ, Johansson MA, Guerra CA, Bhatt S, Golding N, Pigott DM, et al. Modelling adult *Aedes aegypti* and *Aedes albopictus* survival at different temperatures in laboratory and field settings. Parasit Vectors. 2013;6:1–12. 24330720 10.1186/1756-3305-6-351 PMC3867219

[14] Mohammed A, Chadee DD. Effects of different temperature regimens on the development of *Aedes aegypti* (L.) (Diptera: Culicidae) mosquitoes. Acta Trop. 2011;119:38–43. 10.1016/j.actatropica.2011.04.004. 21549680 10.1016/j.actatropica.2011.04.004

[15] Mordecai EA, Cohen JM, Evans MV, Gudapati P, Johnson LR, Lippi CA, et al. Detecting the impact of temperature on transmission of Zika, dengue, and chikungunya using mechanistic models. PLoS Negl Trop Dis. 2017;11:e0005568. 10.1371/journal.pntd.0005568. 28448507 10.1371/journal.pntd.0005568 PMC5423694

[16] Huxley PJ, Murray KA, Pawar S, Cator LJ. The effect of resource limitation on the temperature dependence of mosquito population fitness. Proc Biol Sci. 1949;2021:20203217. 10.1098/rspb.2020.3217. 10.1098/rspb.2020.3217 PMC8079993 33906411

[17] Mackay AJ, Yan J, Kim CH, Barreaux AMG, Stone CM. Larval diet and temperature alter mosquito immunity and development: using body size and developmental traits to track carry-over effects on longevity. Parasit Vectors. 2023;16:434. 10.1186/s13071-023-06037-z. 37993953 10.1186/s13071-023-06037-z PMC10666368

[18] Sharma G, De S, Mandal U, Bhattacherjee R, Suman DS. Ecological variations in adult life table attributes of *Aedes aegypti* (L.) from the desert and coastal regions of India. Med Vet Entomol. 2023;37:164–9. 10.1111/mve.12609. 36070098 10.1111/mve.12609

[19] Hounkanrin G, Tchibozo C, Sauer FG, Agboli E, Schmidt-Chanasit J, Yadouleton A, et al. Genetic diversity and wing geometric morphometrics among four populations of *Aedes aegypti* (Diptera: Culicidae) from Benin. Parasit Vectors. 2023;16:320. 37684701 10.1186/s13071-023-05943-6 PMC10492319

[20] Bennett KL, McMillan WO, Loaiza JR. The genomic signal of local environmental adaptation in *Aedes aegypti* mosquitoes. Evol Appl. 2021;14:1301–13. 10.1111/eva.13199. 34025769 10.1111/eva.13199 PMC8127705

[21] Dennington NL, Grossman MK, Ware-Gilmore F, Teeple JL, Johnson LR, Shocket MS, et al. Phenotypic adaptation to temperature in the mosquito vector, *Aedes aegypti*. Glob Chang Biol. 2024;30:e17041. 10.1111/gcb.17041. 38273521 10.1111/gcb.17041

[22] Sarma DK, Kumar M, Balabaskaran Nina P, Balasubramani K, Pramanik M, Kutum R, et al. An assessment of remotely sensed environmental variables on dengue epidemiology in Central India. PLoS Negl Trop Dis. 2022;16:e0010859. 36251691 10.1371/journal.pntd.0010859 PMC9612820

[23] Hussain SSA, Dhiman RC. Distribution expansion of dengue vectors and climate change in India. Geohealth. 2022;6 6:e2021GH000477; 10.1029/2021GH000477 10.1029/2021GH000477 PMC9210256 35769847

[24] Varamballi P, Babu NN, Mudgal PP, Shetty U, Jayaram A, Karunakaran K, et al. Spatial heterogeneity in the potential distribution of *Aedes* mosquitoes in India under current and future climatic scenarios. Acta Trop. 2024;260:107403. 10.1016/j.actatropica.2024.107403. 39278522 10.1016/j.actatropica.2024.107403

[25] Cosgrove JB, Wood RJ, Petric D, Evans DT, Abbott RH. A convenient mosquito membrane feeding system. J Am Mosq Control Assoc. 1994;10:434–6. 7807091

[26] Masson-Delmotte V, Zhai P, Pirani A, Connors SL, Péan C, Berger S, et al. Climate change 2021: the physical science basis. Contribution of working group I to the sixth assessment report of the intergovernmental panel on climate change. 2021;2:2391.

[27] Dujardin JP. Morphometrics applied to medical entomology. Infect Genet Evol. 2008;8:875–90. 10.1016/j.meegid.2008.07.011. 18832048 10.1016/j.meegid.2008.07.011

[28] Klingenberg CP. MorphoJ: an integrated software package for geometric morphometrics. Mol Ecol Resour. 2011;11:353–7. 10.1111/j.1755-0998.2010.02924.x. 21429143 10.1111/j.1755-0998.2010.02924.x

[29] Marini G, Manica M, Arnoldi D, Inama E, Rosà R, Rizzoli A. Influence of temperature on the life-cycle dynamics of *Aedes albopictus* population established at temperate latitudes: a laboratory experiment. Insects. 2020;11:808. 33212996 10.3390/insects11110808 PMC7698496

[30] Kraemer MUG, Reiner RC Jr, Brady OJ, Messina JP, Gilbert M, Pigott DM, et al. Publisher correction: past and future spread of the arbovirus vectors *Aedes aegypti* and *Aedes albopictus*. Nat Microbiol. 2019;4:901. 10.1038/s41564-019-0440-7. 30962571 10.1038/s41564-019-0440-7 PMC7609323

[31] Carrington LB, Armijos MV, Lambrechts L, Barker CM, Scott TW. Effects of fluctuating daily temperatures at critical thermal extremes on *Aedes aegypti* life-history traits. PLoS ONE. 2013;8:e58824. 23520534 10.1371/journal.pone.0058824 PMC3592833

[32] Brady OJ, Golding N, Pigott DM, Kraemer MU, Messina JP, Reiner RC, Jr., et al. Global temperature constraints on *Aedes aegypti* and *Ae. albopictus* persistence and competence for dengue virus transmission. Parasit Vectors. 2014;7:338. 10.1186/1756-3305-7-338 10.1186/1756-3305-7-338 PMC4148136 25052008

[33] Yang HM, Macoris ML, Galvani KC, Andrighetti MT, Wanderley DM. Assessing the effects of temperature on the population of *Aedes aegypti*, the vector of dengue. Epidemiol Infect. 2009;137:1188–202. 10.1017/S0950268809002040. 19192322 10.1017/S0950268809002040

[34] Ramasamy R, Surendran SN, Jude PJ, Dharshini S, Vinobaba M. Larval development of *Aedes aegypti* and *Aedes albopictus* in peri-urban brackish water and its implications for transmission of arboviral diseases. PLoS Negl Trop Dis. 2011;5:e1369. 10.1371/journal.pntd.0001369. 22132243 10.1371/journal.pntd.0001369 PMC3222631

[35] Jian XY, Jiang YT, Wang M, Jia N, Cai T, Xing D, et al. Effects of constant temperature and daily fluctuating temperature on the transovarial transmission and life cycle of *Aedes albopictus* infected with Zika virus. Front Microbiol. 2022;13:1075362. 10.3389/fmicb.2022.1075362. 36687634 10.3389/fmicb.2022.1075362 PMC9845868

[36] Couret J, Dotson E, Benedict MQ. Temperature, larval diet, and density effects on development rate and survival of *Aedes aegypti* (Diptera: Culicidae). PLoS One. 2014;9:e87468. 10.1371/journal.pone.0087468. 24498328 10.1371/journal.pone.0087468 PMC3911954

[37] Robert MA, Stewart-Ibarra AM, Estallo EL. Climate change and viral emergence: evidence from *Aedes*-borne arboviruses. Curr Opin Virol. 2020;40:41–7. 10.1016/j.coviro.2020.05.001. 32569752 10.1016/j.coviro.2020.05.001 PMC7305058

[38] Scott TW, Amerasinghe PH, Morrison AC, Lorenz LH, Clark GG, Strickman D, et al. Longitudinal studies of *Aedes aegypti* (Diptera: Culicidae) in Thailand and Puerto Rico: blood feeding frequency. J Med Entomol. 2000;37:89–101. 10.1603/0022-2585-37.1.89. 15218911 10.1603/0022-2585-37.1.89

[39] Yeap HL, Endersby NM, Johnson PH, Ritchie SA, Hoffmann AA. Body size and wing shape measurements as quality indicators of *Aedes aegypti* mosquitoes destined for field release. Am J Trop Med Hyg. 2013;89:78. 23716403 10.4269/ajtmh.12-0719 PMC3748492

[40] Alto BW, Reiskind MH, Lounibos LP. Size alters susceptibility of vectors to dengue virus infection and dissemination. Am J Trop Med Hyg. 2008;79:688. 18981505 PMC2630770

[41] Alto BW, Lounibos LP, Higgs S, Juliano SA. Larval competition differentially affects arbovirus infection in *Aedes* mosquitoes. Ecology. 2005;86:3279–88. 10.1890/05-0209. 19096729 10.1890/05-0209 PMC2605070

[42] Sharma G, Sarma DK, Bhutiani R. Effect of environmental conditions on the wing morphometric variation in *Aedes aegypti* (Diptera: Culicidae) in India. bioRxiv. 2024:2024.02. 01.578359.

[43] Sarma DK, Rathod L, Mishra S, Das D, Agarwal A, Sharma G, et al. Molecular surveillance of dengue virus in field-collected *Aedes* mosquitoes from Bhopal, Central India: evidence of circulation of a new lineage of serotype 2. Front Microbiol. 2023; 14:1260812; 10.3389/fmicb.2023.1260812. 10.3389/fmicb.2023.1260812 PMC10539573 37779723

[44] Yan J, Kim C-H, Chesser L, Ramirez JL, Stone CM. Nutritional stress compromises mosquito fitness and antiviral immunity, while enhancing dengue virus infection susceptibility. Commun Biol. 2023;6:1123. 37932414 10.1038/s42003-023-05516-4 PMC10628303

