# Supplementary material for: Thermal influence on development and morphological traits of Aedes aegypti in central India and its relevance to climate change
Source: Parasit Vectors. 2025 Jul 11;18:279. doi: 10.1186/s13071-025-06924-7 (PMC12255045; doi:10.1186/s13071-025-06924-7)
Supplement: Supplementary file 1 — Additional File 1: Supplementary Text S1. Codes for calculation of future temperature projections. [file 13071_2025_6924_MOESM1_ESM.docx]

**Text S1. Codes for Calculation of future temperature projections**

import pandas as pd
import matplotlib.pyplot as plt
from statsmodels.tsa.statespace.sarimax import SARIMAX
from sklearn.metrics import mean_squared_error
import numpy as np
import xarray as xr

# Upload the file to Google Colab
from google.colab import files
uploaded = files.upload()

# Read the Excel file
data = pd.read_excel(list(uploaded.keys())[0])

# Parse the date column and set it as index
data['Date'] = pd.to_datetime(data['Date'], format='%d/%m/%Y')
data.set_index('Date', inplace=True)

# Resample data to yearly averages
yearly_data = data.resample('Y').mean()

# Plotting the historical data
def plot_historical(data, variable, ylabel):
    plt.figure(figsize=(12, 6))
    plt.plot(data.index, data[variable], label=f'Historical {variable}')
    plt.title(f'Yearly Average {variable} (Historical)', fontsize=14)
    plt.xlabel('Year', fontsize=12)
    plt.ylabel(ylabel, fontsize=12)
    plt.grid()
    plt.legend()
    plt.show()

for var in ['T2M_MIN', 'T2M_MAX', 'T2M']:
    plot_historical(yearly_data, var, ylabel=f'{var} (°C)')

# Forecasting using ARIMA
def forecast_arima(data, variable, forecast_years):
    # Fit SARIMAX model
    model = SARIMAX(data[variable], order=(2, 1, 1), seasonal_order=(0, 1, 2, 12))
    results = model.fit(disp=False)

    # Forecast
    forecast = results.get_forecast(steps=forecast_years)
    forecast_index = pd.date_range(start=data.index[-1] + pd.DateOffset(years=1),
                                   periods=forecast_years, freq='Y')
    forecast_mean = forecast.predicted_mean
    forecast_ci = forecast.conf_int()

    # Plot forecast
    plt.figure(figsize=(12, 6))
    plt.plot(data.index, data[variable], label=f'Historical {variable}')
    plt.plot(forecast_index, forecast_mean, color='orange', label='Forecast')
    plt.fill_between(forecast_index,
                     forecast_ci.iloc[:, 0],
                     forecast_ci.iloc[:, 1],
                     color='blue', alpha=0.2, label='Confidence Interval')
    plt.title(f'Forecast of {variable} (Yearly)', fontsize=14)
    plt.xlabel('Year', fontsize=12)
    plt.ylabel(f'{variable} (°C)', fontsize=12)
    plt.grid()
    plt.legend()
    plt.show()

    return forecast_mean

# Predict until 2100
forecast_years = 2100 - yearly_data.index[-1].year
forecast_results = {}

for var in ['T2M_MIN', 'T2M_MAX', 'T2M']:
    print(f'Forecasting for {var}...')
    forecast_results[var] = forecast_arima(yearly_data, var, forecast_years)

# Generate synthetic SSP projections
def generate_synthetic_ssp(historical_data, variable, scenario_name, forecast_years, growth_rate):
    """
    Generates synthetic SSP projections based on historical data and a growth rate.
    Args:
        historical_data (pd.DataFrame): Historical data for the variable.
        variable (str): Variable to project (e.g., T2M, T2M_MIN, T2M_MAX).
        scenario_name (str): Scenario name (e.g., SSP1-2.6).
        forecast_years (int): Number of years to forecast.
        growth_rate (float): Annual growth rate for the projection.
    Returns:
        pd.DataFrame: Synthetic SSP projection.
    """
    last_value = historical_data[variable].iloc[-1]
    future_years = pd.date_range(start=historical_data.index[-1] + pd.DateOffset(years=1),
                                 periods=forecast_years, freq='Y')
   
    # Apply growth rate to generate projections
    synthetic_projection = [last_value * ((1 + growth_rate) ** i) for i in range(forecast_years)]
   
    ssp_data = pd.DataFrame({
        'Date': future_years,
        'Value': synthetic_projection
    })
    ssp_data.set_index('Date', inplace=True)
    return ssp_data

# Define growth rates for SSP scenarios
ssp_scenarios = {
    'SSP1-2.6': 0.002,  # Very low growth rate
    'SSP2-4.5': 0.004,  # Moderate growth rate
    'SSP5-8.5': 0.007   # High growth rate
}

# Generate SSP projections for each variable
synthetic_ssp_projections = {var: {} for var in ['T2M_MIN', 'T2M_MAX', 'T2M']}
for var in ['T2M_MIN', 'T2M_MAX', 'T2M']:
    for scenario, rate in ssp_scenarios.items():
        synthetic_ssp_projections[var][scenario] = generate_synthetic_ssp(yearly_data, var, scenario, forecast_years, rate)

# Plot historical data, ARIMA forecast, and SSP projections
def plot_all_projections(historical_data, forecast_data, ssp_data_dict, variable):
    plt.figure(figsize=(12, 6))
   
    # Plot historical data
    plt.plot(historical_data.index, historical_data[variable], label=f'Historical {variable}', color='black')
   
    # Plot ARIMA forecast
    if forecast_data is not None:
        plt.plot(forecast_data.index, forecast_data, label='ARIMA Forecast', color='orange')
   
    # Plot SSP projections
    for scenario, data in ssp_data_dict.items():
        plt.plot(data.index, data['Value'], label=f'{scenario} Projection')
   
    plt.title(f'{variable} Projections (Historical, Forecast, and SSPs)', fontsize=14)
    plt.xlabel('Year', fontsize=12)
    plt.ylabel(f'{variable} (°C)', fontsize=12)
    plt.grid()
    plt.legend()
    plt.show()

# Plot projections for all variables
for var in ['T2M_MIN', 'T2M_MAX', 'T2M']:
    plot_all_projections(yearly_data, forecast_results[var], synthetic_ssp_projections[var], variable=var)
